# Debriefer cognitive load during Traditional Reflective Debriefing vs. Rapid Cycle Deliberate Practice interdisciplinary team training

**DOI:** 10.1186/s41077-024-00296-1

**Published:** 2024-06-04

**Authors:** Susan Wiltrakis, Ruth Hwu, Sherita Holmes, Srikant Iyer, Nandranie Goodwin, Claire Mathai, Scott Gillespie, Kiran B. Hebbar, Nora Colman

**Affiliations:** 1 Division of Emergency Medicine, Department of Pediatrics, Washington University in St. Louis, 660 S. Euclid Ave, St. Louis, MO 63110 USA; 2 Division of Emergency Medicine, Department of Pediatrics, Emory University School of Medicine, Atlanta, GA USA; 3 Children’s Healthcare of Atlanta, Atlanta, GA USA; 4 Department of Biostatistics, Emory University School of Medicine, Atlanta, GA USA; 5 Division of Critical Care Medicine, Department of Pediatrics, Emory University School of Medicine, Atlanta, GA USA

**Keywords:** Cognitive Load Theory, Facilitator cognitive load, Interdisciplinary simulation education

## Abstract

**Background:**

Cognitive load impacts performance of debriefers and learners during simulations, but limited data exists examining debriefer cognitive load. The aim of this study is to compare the cognitive load of the debriefers during simulation-based team training (SbTT) with Rapid Cycle Deliberate Practice (RCDP) debriefing and Traditional Reflective Debriefing (TRD). We hypothesize that cognitive load will be reduced during RCDP compared to TRD.

**Methods:**

This study was part of a large-scale, interdisciplinary team training program at Children’s Healthcare of Atlanta Egleston Pediatric Emergency Department, with 164 learners (physicians, nurses, medical technicians, paramedics, and respiratory therapists (RTs)). Eight debriefers (main facilitators and discipline-specific coaches) led 28 workshops, which were quasi-randomized to either RCDP or TRD. Each session began with a baseline medical resuscitation scenario and cognitive load measurement using the NASA Task Load Index (TLX), and the NASA TLX was repeated immediately following either TRD or RCDP debriefing. Raw scores of the NASA TLX before and after intervention were compared. ANOVA tests were used to compare differences in NASA TLX scores before and after intervention between the RCDP and TRD groups.

**Results:**

For all debriefers, mean NASA TLX scores for physical demands and frustration significantly decreased (− 0.8, *p* = 0.004 and − 1.3, *p* = 0.002) in TRD and mean perceived performance success significantly increased (+ 2.4, *p* < 0.001). For RCDP, perceived performance success increased post-debriefing (+ 3.6, *p* < 0.001), time demands decreased (− 1.0, *p* = 0.04), and frustration decreased (− 2.0, *p* < 0.001). Comparing TRD directly to RCDP, perceived performance success was greater in RCDP than TRD (3.6 vs. 2.4, *p* = 0.04). Main facilitators had lower effort and mental demand in RCDP and greater perceived success (*p* < 0.001).

**Conclusion:**

RCDP had greater perceived success than TRD for debriefers. Main facilitators also report reduced effort and baseline mental demand in RCDP. For less experienced debriefers, newer simulation programs, or large team training sessions such as our study, RCDP may be a less mentally demanding debriefing methodology for facilitators.

## Background

In an effort to design successful simulation experiences in healthcare education, learners’ abilities to successfully acquire, transfer, and apply knowledge during simulation-based education has been the focus of prior research [1]. According to Cognitive Load Theory (CLT), learning can only occur if there is adequate working memory [2–4]. From the perspective of the learner, simulation scenarios are often complex, and extraneous cognitive load can overwhelm the working memory capacity, impacting performance and learning [4–6]. Debriefing is a critical component of simulation success, with a wide variety of approaches described in simulation literature [7]; while some debriefing techniques focus on reflective learning, others provide more directive feedback in performance and knowledge gaps.

Two common debriefing approaches applied in education-based simulation are Traditional Reflective Debriefing (TRD), using reflection-on-action, and Rapid Cycle Deliberate Practice (RCDP), focusing on directed feedback and repetitive practice [8, 9]. The role of the debriefer is a challenging one, and techniques such as advocacy-inquiry and directive feedback require practice to achieve mastery and maintain proficiency [7, 10]. Debriefers experience high cognitive load as they juggle multiple tasks simultaneously; observing then recalling simulation events, effectively navigating learning objectives, encouraging learner engagement, all while managing learner emotions and protecting learner psychological safety [7]. Three types of cognitive load that are involved in debriefing have been described: intrinsic cognitive load (recalling simulation events and listening to learner responses), extraneous load (performing tasks outside the debriefing, such as mannequin management), and germane load (learning from experience, such as processing successful or unsuccessful approaches during debriefing and adjusting to the learners’ responses). In addition to the cognitive load described above, there is a significant workload in the execution of debriefing skills and processing [10]. This unique combination of cognitive load and workload from execution of tasks involved with effective debriefing is taxing and is becoming an active area of study.

While the impact of learner cognitive load during simulation has been widely investigated, there is limited data regarding the cognitive load of debriefers during simulation and debriefing, and furthermore, no research exists comparing debriefer cognitive load between different debriefing methodologies [1, 3, 6]. In this study, we evaluated the impact of two different debriefing methods (RCDP and TRD) on the workload of debriefers during interdisciplinary simulation-based team training (SbTT). RCDP lends an opportunity to decrease the intrinsic cognitive load through directed feedback and repetitive practice approach, potentially minimizing the facilitator’s need to remember and recall specific events. Thus, we hypothesize debriefer cognitive load and workload of executing debriefing would be lower in RCDP compared to TRD.

## Methods

### Study design

**Fig. 1:**
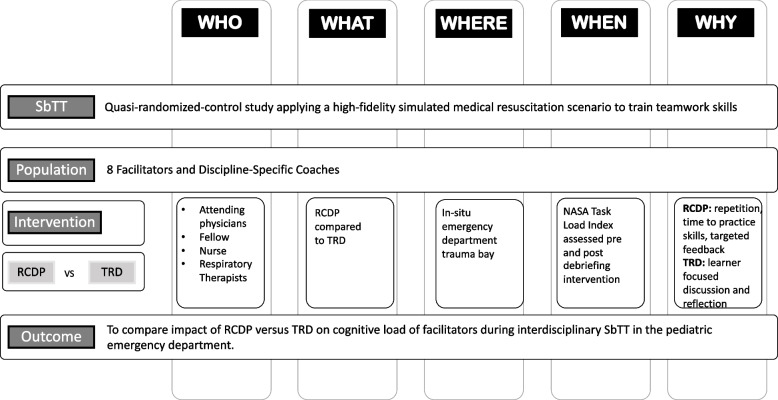
Facilitator and coach cognitive load study design: RCDP vs. TRD

**Fig. 2:**
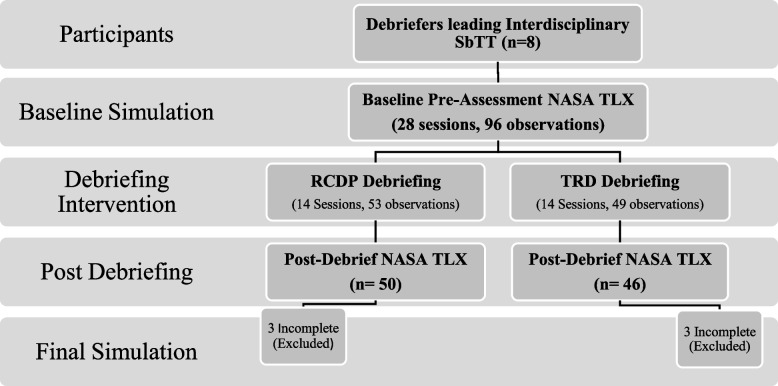
Study design

This was a prospective, un-blinded, quasi-randomized control study comparing cognitive load of debriefers leading SbTT sessions using RCDP or TRD debriefing (Fig. 1). From October to November 2019, debriefers led twenty-eight 3-h workshops for pediatric emergency medicine (PEM) staff. Groups were quasi-randomized to either RCDP or TRD debriefing based on date of the session; participants signed up for sessions without knowledge of the assigned debriefing method. The primary outcome included cognitive load of debriefers leading SbTT sessions. Cognitive load and perceived workload were measured by self-reporting using the National Aeronautics and Space Administration Task Load Index (NASA TLX) [10] (Appendix 1 in Table 3). Cognitive load of the debriefers were measured after the baseline simulation at the start of the workshop and after debriefing intervention with RCDP or TRD, and the learners completed on final simulation without interruption (Fig. 2). Team training was mandatory for the PEM Department, and learners and debriefers consented to be part of the research study. This study was approved by the Emory Institutional Review Board.

### Setting

SbTT sessions were conducted in the Children’s Healthcare of Atlanta (CHOA) Emergency Department at Egleston. Trauma resuscitation rooms were used, or an alternate ED room if otherwise occupied. A high-fidelity human child mannequin (Gaumard Hal S157, 5 year old) with capabilities including heart and lung sounds, palpable pulses, and two functional intravenous (IV) ports was utilized. Simulation equipment embedded in the trauma resuscitation room included IV fluids and tubing, mock code drug tray, defibrillator and pads, stools, backboard, and airway equipment. Debriefings took place in the room used for the simulation.

### Debriefers

For each 3 hour simulation workshop, one main facilitator, one simulation technician, and a minimum of three coaches (nursing, physician, and respiratory therapist (RT)) participated. Facilitators were either pediatric critical care or emergency medicine attendings, with extensive debriefing experience. Coaches consisted of pediatric critical care or emergency medicine physicians (attending or fellow), as well as experienced nurse and RT educators, with prior debriefing training. For purposes of analysis, main facilitator and coach data were combined, and this group was referred to as “debriefers.” All debriefers had training in TRD and RCDP and both methodologies used commonly in institutional simulation sessions.

### Learners

Learner participants for each session included one pediatric emergency medicine (PEM) attending, one PEM fellow as available, two or three nurses, two paramedics or medical technicians, and one respiratory therapist (RT). All learners worked in the Pediatric ED at Children’s Healthcare of Atlanta (CHOA) at Egleston. PEM attending physicians served as team leader, and if present, PEM fellows served as the second physician managing airway. An embedded coach otherwise served as the airway physician. Nursing roles included documentation, primary bedside nurse, and medication nurse (responsible for drawing up medications). Each learner stayed in their discipline-specific role during the simulation and received feedback from debriefers.

### Intervention and outcomes

#### Simulated scenario

The same simulated scenario was used for both TRD and RCDP session: a 6-year-old patient presenting in undifferentiated shock. The patient developed progressive uncompensated shock characterized by tachycardia, worsening hypotension, and respiratory failure, requiring endotracheal intubation and ultimately progressing to cardiac arrest, regardless of learner interventions. The scenario was pre-programmed, and debriefers were provided a detailed script with learning objectives for TRD and pre-determined hard and soft stops for RCDP. Debriefers led SbTT workshops with either TRD or RCDP, depending on session date. During the baseline simulation in both debriefing methods, learners completed the scenario without any interruptions or feedback. Following the baseline scenario, TRD or RCDP debriefing was led by debriefers.

#### TRD debriefing

TRD took place over 60 to 70 minutes with three phases: reaction, descriptive, and analysis phases. Learning objectives focused on teamwork and communication skills (specifically closed loop communication, directed communication, role assignment, and role clarity). Debriefing sessions were led by the main facilitator and structured based on PEARLS debriefing strategy, utilizing plus-delta and advocacy-inquiry techniques [8]. Coaches co-facilitated by assessing the learners’ frames, providing discipline-specific feedback, and helping close performance gaps for learners. After the debriefing session, the learners repeated the scenario to practice skills and concepts discussed during the debriefing session.

#### RCDP debriefing

Following the baseline simulation that was completed without interruption, 60 minutes was allotted for RCDP debriefing. Scenario learning objectives were determined for each phase of the scenario (uncompensated shock, intubation, and cardiac arrest). During RCDP debriefing, the learners repeated the scenario while the debriefers observed the learners and paused the entire team to provide feedback and coaching based on the predetermined hard and soft stops related to teamwork (role identification, shared mental model, closed loop communication, etc.) and cardio-respiratory resuscitation concepts (backboard placement, epinephrine timing, CPR coaching, etc.). During the pauses, the main facilitator provided generalized feedback to the team, while the nurse, physician, and RT coaches provided discipline directed feedback to their respective learners (physicians coached physicians, nurses coached nurses, etc.). The main facilitator directed repetition and rewinding of the scenario based on the teams’ ability to achieve the learning objectives. If the team successfully performed the learning objective, positive feedback was given, and the scenario progressed. The scenario was then run a final time without feedback or interruption.

### Data collection

Data was collected using Redcap, an online survey program. Debriefers were de-identified prior to analysis.

Demographic information of debriefers was collected using a survey at the start of each SbTT workshop. Cognitive load and workload were measured using the NASA TLX (10, Appendix 1 in Table 3). While the NASA TLX was initially used in aviation, it has been validated to measure mental workload in healthcare and simulation [11–13]. This tool is a six-item scale that measures mental demand, physical demand, temporal demand, effort, performance, and frustration [5, 12]. This scale was converted to a 10-point Likert scale, with a scale ranging from 0 to 10 for each component of the six-question survey. NASA TLX was completed by debriefers following the baseline simulation at the start of the SbTT session, and a second NASA TLX survey was completed immediately following debriefing with RCDP or TRD.

### Statistical analysis

Demographics of debriefers, including age, sex, profession, work experience, and prior simulation experience, were summarized using counts and percentages. NASA TLX scores were summarized pre and post intervention using means and standard deviations, and inferentially assessed using mixed effects two-factor ANOVA. In the mixed effects ANOVA models, the fixed effects were time (pre vs. post intervention), study group (RCDP vs. TRD), and the 2-way interaction of time by study group; random effects were the participant-level intercepts. Within study group estimates from the mixed effects ANOVA models were least squares (LS) mean differences from pre to post, 95% confidence intervals and *p* values, and effects sizes (ES). ES were calculated by dividing LS mean differences by their respective baseline standard deviation and interpreted as small (0.2), moderate (0.5), and large (0.7). Differences between the study groups were evaluated by the significance of the 2-way interaction term *p* values. Additionally, moderator analyses were performed, examining debriefer years of experience in current position (< 5 years vs. > 5 years), again using mixed effect ANOVA model estimates. Specifically, 3-way interactions were first modeled to evaluate pre-post changes in outcomes between TRD and RCDP participants varied by levels of the moderators. Next, the full study sample was stratified by levels of the moderator variables, and 2-way interaction *p* values were similarly calculated and interpreted, as in the overall analysis. All analyses were performed in SAS v. 9.4 (Cary, NC), and statistical significance was evaluated at the 0.05 level.

## Results

Eight debriefers conducted twenty-eight session, consisting of one hundred and sixty-four learners (Table 1). All were female and had prior simulation training and team-based simulation training. Most had been in their current position less than 5 years.

**Table 1:** Demographics (*n* = 8)

**Female gender**	**8 (100%)**
**Profession**	
**Attending physician**	3 (37%)
**Fellow**	1 (13%)
**Nurse**	1 (13%)
**Respiratory therapist**	3 (37%)
**Age (years)**	
**18-29**	2 (25%)
**30-39**	4 (50%)
**40-49**	1 (12.5%)
**50 + **	1 (12.5%)
**Time in current position (years)**	
** < 2**	1 (12.5%)
**2-5**	4 (50%)
**6-10**	0 (0%)
**11-15**	1 (12.5%)
** > 15**	2 (25%)
**Previous simulation training**	
** > 10 years**	8 (100%)
**Prior team-based training—yes**	8 (100%)

For all debriefers using TRD, mean NASA TLX scores for physical demands and frustration significantly decreased post-debriefing (− 0.76, *p* = 0.004 and − 1.26, *p* = 0.002, respectively, Table 2), while mean NASA TLX score for perceived performance success significantly increased post-debriefing (+ 2.39, *p* < 0.001, Table 2). For all debriefers using RCDP, mean NASA TLX score for perceived performance success increased post-debriefing (+ 3.6, *p* < 0.001, Table 2) and mean NASA TLX score for time demands and frustration decreased (− 1.0, *p* = 0.037, − 2.02, *p* < 0.001, respectively, Table 2).

**Table 2:** Mean NASA TLX scores for all debriefers (*n* = 8)

**NASA TLX question**	**Baseline** **mean ± SD**	**Post debrief** **mean ± SD**	**LS-mean difference (95% CI)**	**Post–pre** ***p* value (ES)**
**Mental demand**				
TRD (*N* = 46 obs.)	6.65 ± 1.70	6.80 ± 1.90	0.15 (-0.39, 0.69)	0.579 (0.09)
RCDP (*N* = 50 obs.)	5.76 ± 2.10	5.56 ± 1.83	-0.20 (-0.72, 0.32)	0.448 (0.10)
**Physical demand**				
TRD (*N* = 46 obs.)	3.46 ± 2.80	2.70 ± 2.32	-0.76 (-1.28, -0.24)	0.004* (0.27)
RCDP (*N* = 50 obs.)	3.46 ± 2.43	3.38 ± 1.96	-0.08 (-0.58, 0.42)	0.749 (0.03)
**Time demand**				
TRD (*N* = 46 obs.)	4.35 ± 2.63	3.96 ± 2.13	-0.39 (-1.37, 0.59)	0.429 (0.15)
RCDP (*N* = 50 obs.)	4.50 ± 2.71	3.50 ± 1.92	-1.00 (-1.94, -0.06)	0.037* (0.37)
**Performance**				
TRD (*N* = 46 obs.)	4.83 ± 2.68	7.22 ± 2.12	2.39 (1.57, 3.21)	< 0.001* (0.89)
RCDP (*N* = 50 obs.)	4.34 ± 2.54	7.94 ± 1.71	3.60 (2.81, 4.39)	< 0.001* (1.42)
**Effort**				
TRD (*N* = 46 obs.)	5.41 ± 2.53	5.87 ± 2.69	0.46 (-0.33, 1.24)	0.250 (0.18)
RCDP (*N* = 50 obs.)	4.80 ± 2.35	5.04 ± 2.11	0.24 (-0.51, 0.99)	0.528 (0.10)
**Frustration**				
TRD (*N* = 46 obs.)	4.30 ± 2.76	3.04 ± 2.28	-1.26 (-2.05, -0.47)	0.002* (0.46)
RCDP (*N* = 50 obs.)	4.24 ± 2.74	2.22 ± 1.75	-2.02 (-2.78, -1.26)	< 0.001* (0.74)

^*^Statistically significant

**Fig. 3:**
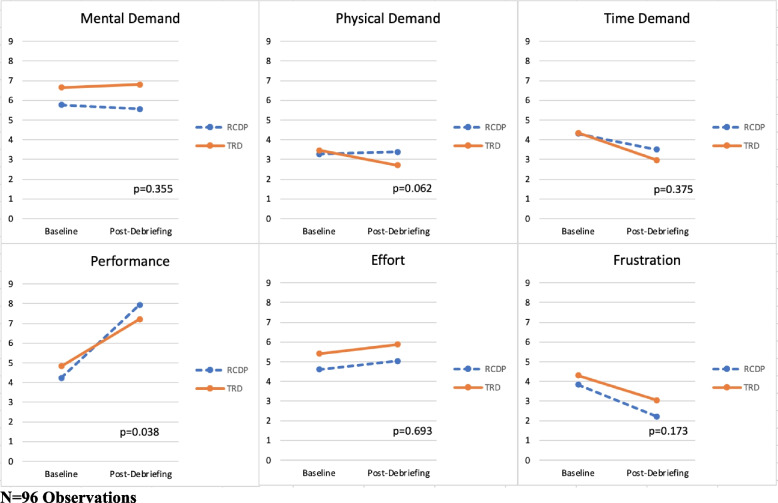
TRD and RCDP NASA TLX LS-mean score difference

When comparing TRD directly to RCDP using LS-mean score difference, RCDP had greater perceived success compared to TRD (3.60 vs. 2.39, *p* = 0.038) (Fig. 3). However, changes in mental demand, physical demand, time demand, effort, and frustration were not significant between RCDP and TRD.

Moderator analysis was completed to evaluate the impact of debriefer experience on cognitive load. There were no significant differences in cognitive load of more experienced (> 5 years in current position) compared to less experienced (< 5 years in current position) during RCDP vs. TRD. However, trends included less experienced debriefers reporting lower mental demand and effort with RCDP compared with more experienced debriefers (mean post-debriefing score mental demand: 5 vs. 6.87, effort 4.63 vs. 6.0, Appendix 2 in Table 4).

**Fig. 4:**
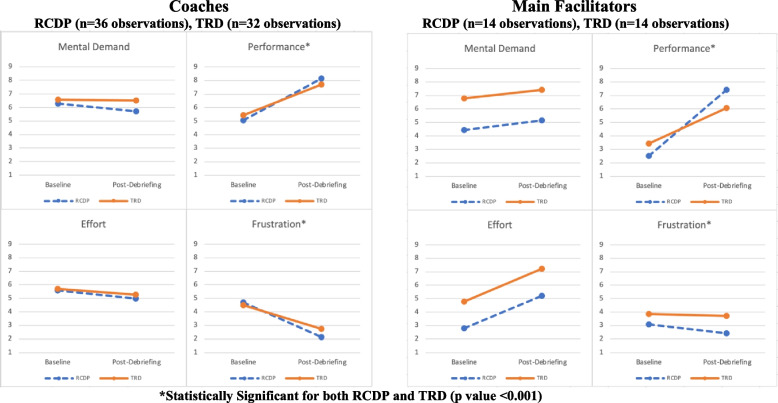
NASA TLX scores TRD vs. RCDP for coaches and main facilitators

In the debriefer subgroup analysis individually evaluating main facilitators and coaches, main facilitators reported greater perceived performance in RCDP vs. TRD (*p* = 0.017), and trends included higher effort in TRD compared to RCDP (Fig. 4). Post-debriefing mental demand scores were also higher in TRD compared with RCDP for main facilitators (7.43 vs. 5.14), but the change in score was not statistically significant. For coaches, there was no significant change in effort or mental demand between TRD and RCDP (Fig. 4). Coaches reported decreased frustration in both TRD and RCDP (mean score change − 1.75 (*p* = 0.001) and − 2.56 (*p* < 0.001), respectively), while the main facilitator’s frustration was nearly unchanged in TRD or RCDP (− 0.14 (*p* = 0.788) and − 0.64 (*p* = 0.233), Fig. 4).

## Discussion

Debriefer cognitive load and high mental demands directly impact the quality of debriefing, in turn influencing the overall success of the simulation and learner experience. For all debriefers, RCDP was perceived as more successful, less hurried, and less frustrating. For main facilitators, TRD had higher effort and mental demand scores, while RCDP was perceived as more successful. Debriefer prior experience did not have an impact on cognitive load in either debriefing methodology.

For all debriefers, RCDP was perceived as a more successful debriefing methodology than TRD for SbTT (*p* = 0.038). This success is likely multifactorial, but due in large part to the deliberate practice component of RCDP. Learners continued to practice skills until mastery was demonstrated to the debriefers. For example, read back and verification (RBAV) surrounding medication delivery was not routinely performed by learners in the baseline simulations. This skill is critical to help reduce medication errors but was not a standard practice for many learners. The immediate application during RCDP and repeated practice at multiple points throughout the scenario not only gave learners an opportunity to learn this skill, but also demonstrated the positive impact RBAV had on team communication and scenario success. Additionally, debriefers were able to witness the learners demonstrating this new skill during the RCDP cycles until mastery was achieved. In TRD, the learners did not have the same opportunity to practice and demonstrate their skills multiple times following feedback. While great discussions regarding the barriers to RBAV occurred during TRD (including fear of interrupting the physician and feeling time pressured), the learners only had the final simulation to run-through RBAV, and thus were not able to practice or demonstrate this skill to the same degree as learners in RCDP.

Discipline-specific coaches may represent another factor contributing to superior perceived success of RCDP. The coaches played a more active role in RCDP, providing tailored, immediate feedback to their respective learner groups. By contrast, in TRD, this was led mainly by the main facilitator. The coach had similar background to their learner group, which we believe helped minimize reluctance to adopt new skills or behaviors. The content of our SbTT workshop focused on communication skills, which can be challenging to debrief, as they are deeply rooted in institution culture and habits. There may be resistance or hesitation by learners to participate in discussion with a facilitator who has a different background and is not part of their discipline. Discipline-specific coaches may have more easily established psychological safety, as the coaches understood the unique perspective and challenges of the learners. One example of this during the SbTT sessions was communication challenges between the physician and RT prior to intubation. We observed that intubation often proceeded before the RT was fully prepared. The RT coach was able to provide credible feedback to the RT learner, since they understand both the time pressures and nuances of the workflows integral for intubation preparation. It became clear that the RT would become so fixated on gathering and preparing supplies for intubation that they missed physician orders that intubation was proceeding. The RT coach shared their perspective to help close the communication gap, made note the importance of breaking the target fixation and listening for the physicians shared mental model regarding intubation readiness, and suggested the most effective timing for the learner to share their mental model. This shared understanding facilitated learner buy-in, which may not have occurred if feedback was provided by a facilitator of a different discipline. The structure of RCDP allowed the learner to practice this skill compared with TRD that was able to repeat the scenario but without directed feedback or practice during the prior facilitation. With this additional practice, the other learners in the team gained appreciation and understanding of the RTs role in intubation preparation.

The detailed scripting with discrete hard and soft stops in RCDP debriefing session helped provide structure and likely minimized intrinsic cognitive load for debriefers. The complex scenario was divided into discrete phases and specific learning objectives for each discipline were anchored to each phase. This allowed the debriefers to move through the scenario without having to watch and synthesize the simulation in entirety. A detailed debriefing guide with hard and soft stops for each phase was provided to all debriefers, providing a predictable roadmap for how the debriefing would unfold. For example, role assignment and leader identification were always covered at the beginning of the scenario at the first hard stop, whereas directed communication was the focus during the decompensation phase of the scenario. The debriefers had a shared mental model regarding the order that learning objectives would be addressed, and repetitive facilitation of the same training session also hardwired debriefing so that it became second-nature for debriefers with each subsequent session. In contrast, with TRD, the learner-driven discussion was less predictable and thus the facilitators and coaches may compete to cover learning objectives or cover learning objectives in a different order during each debriefing session. The learner-driven approach also potentially led to time devoted to other discussions not completely focused on pre-determined the learning objectives.

In both debriefing methodologies, we propose that extraneous cognitive load was decreased by the presence of a simulation technologist and coaches, but especially emphasized in RCDP. In prior studies evaluating cognitive load of facilitators, one study found that the addition of simulation technologists removed some extraneous load for debriefers and decreased cognitive load [6], but impact of debriefing methodology was not examined. While simultaneously managing the list of hard and soft stops in RCDP and watching the simulation could be overwhelming, the presence of multiple debriefers helped distribute the mental effort and minimize the intrinsic cognitive load for debriefers. This was most dramatically demonstrated for the main facilitator, as evidenced by lower frustration and mental load scores for RCDP.

Mental workload, level of frustration, and effort required to achieve perceived success were particularly high for main facilitators during TRD. This is likely related to the nature of TRD, debriefing experience, learner demographics, and the subject of simulation training. High baseline mental workload (6.79 in TRD vs. 4.43 in RCDP) was related to the demanding nature of observing and taking in the entire simulated scenario during TRD. The large interdisciplinary SbTT consisted of multiple simulation phases with complex, dynamic interactions occurring simultaneously throughout the scenario. In TRD sessions, the debriefer had to watch the scenario in entirety, make note of behaviors that met or failed to meet learning objectives, while also facilitating the scenario, guiding the participants to keep the scenario on track, and providing clinical or historical information when prompted by learners. At the completion of the scenario, there was a short time to formulate thoughts, summarize the events of the scenario, and generate a plan for debriefing. In contrast to RCDP, the baseline scenario in TRD served as an introduction for the facilitator to gauge areas of strength and weakness in the learners and begin mapping the debriefing. The increase in mental workload for the main facilitator after TRD (post score of 7.4) reflects the cognitive load required to guide a productive debriefing. In real time, the debriefer listened, synthesized, quote/paraphrased, and interpreted the learner’s reactions to generate a learner-centered discussion to challenge embedded assumptions, level perceptions across disciplines, and close performance gaps. While PEARLS debriefing provides a scripted guide for facilitators to minimize cognitive load, establishing physiological safety, eliciting learners’ frame, delving into learner perception and perspectives, and providing focused feedback to close knowledge or performance gaps still generate significant cognitive load.

Our results can be generalizable to other large-scale simulation initiatives, specifically focused on team training. The generalizability of this study to SbTT with only one debriefer may be limited. The focus of the SbTT sessions was mainly communication and teamwork skills, but medical knowledge learning objectives were also integrated (effective cardiopulmonary resuscitation and rapid-sequence intubation). The interdisciplinary nature of the learner population added another layer of complexity for the debriefing team, but we speculate that the presence of discipline-specific coaches helped reduce extraneous and intrinsic cognitive load of the facilitator. While debriefer workload and cognitive load in learning debriefing skills are important considerations in simulation session planning, there are many other factors to consider, including content, learner group, and time allotment for learning session. Additionally, the goals of the sessions may assist in selection the most appropriate debriefing methodology; for instance, skill-based or algorithm-based topics such as cardiac arrest management lend themselves to the RCDP model, as metrics and performance expectations are clear and more universal [9]. Alternatively, more complex communication skills or processes would lend themselves to TRD, as understanding frames and cultural and hierarchical needs are essential in driving performance changes [8].

The two debriefing strategies highlighted in this study not only result in different facilitation and debriefing styles for debriefers, but also differences in learner experience. It is important to not only consider the workload of debriefers when selecting debriefing strategy, but also consider learner experiences, learning objectives, and goals of the simulation session. However, it is important to remember many factors contribute to cognitive load—learner interactions, simulation environment, learning objective topics, and prior debriefing experience. Next steps include examining learner cognitive load and the impact that different learner groups may have on debriefer cognitive load; specifically, the impact learner demographics and prior experience, in addition to investigating how debriefer cognitive load is correlated with learner-perceived success during SbTT.

Limitations of this study include a small and homogenous debriefer group. Eight individuals led simulation sessions, and while all had prior debriefing training, most were relatively new to their position (majority having less than 5 years in their current position). The study was not powered to separate facilitators and coaches from each other, due to small sample size. All debriefers are from the same institution and had training but varying experience with RCDP prior to simulation sessions, which could contribute to perceived workload variation. However, due to the younger, less experienced debriefer group, RCDP may have been positively received, due to capacity to distribute responsibility among debriefers and divide learning objectives into more manageable units. Another limitation of our study involves the timing of the NASA TLX survey. The cognitive load of debriefers was only measured at the end of debriefing, not during debriefing. This may be missing fluctuations in cognitive load and mental demands during the debriefing activity. Additionally, learner group characteristics may influence the demands of the debriefing. While learner group demographics were not significantly different between TRD and RCDP groups, small differences in learner group experience and composition may influence cognitive load of the debriefers. It is important to note that RCDP by nature has repeated aspects of the scenario, which may reinforce debriefers’ memory and reduce workload and could have impacted debriefer responses in this study. A consideration for future studies will be a direct comparison of dual cycle TRD and RCDP, with scripting for both debriefing methodologies, which may allow for a more direct comparison of outcomes.

## Conclusions

In conclusion, RCDP had greater perceived success than TRD for all debriefers. This may indicate that RCDP is easier to facilitate and represented a debriefer preference. In this sample, TRD may be more challenging for both the debriefer, as evidenced by higher mental demand and effort for main facilitators; since learning objectives are woven into the discussion (driven by learner reflection), there may be increased intrinsic cognitive load. For less experienced debriefers, newer simulation programs, or large team training sessions with multiple debriefers, such as in our study, RCDP may be a less mentally taxing and preferred methodology for debriefers.

## Data Availability

All data generated or analyzed during this study are included in this published article [and its supplementary information files].

## Appendix 1

**Table:** NASA TLX questions (scale of 1–10)

	**Category**	**Question**
Question 1	Mental demand	How mentally demanding was the task?
Question 2	Physical demand	How physically demanding was the task?
Question 3	Time demand	How hurried or rushed was the pace of the task?
Question 4	Performance	How successful were you in accomplishing what you were asked to do?
Question 5	Effort	How hard did you have to work to accomplish your level of performance?
Question 6	Frustration	How insecure, discouraged, irritated, stressed, and annoyed were you?

## Appendix 2

**Table:** NASA TLX for facilitators, by years in current position

NASA	Pre mean ± SD	Post mean ± SD	LS-mean post–pre difference (95% CI)	Post–pre *p* value (ES)
**Mental demand**				
** ≤ 5 years current position**				
TRD (*N* = 28 obs.)	6.07 ± 1.41	6.46 ± 1.71	0.39 (-0.34, 1.12)	0.286 (0.28)
RCDP (*N* = 35 obs.)	5.14 ± 2.03	5.00 ± 1.48	-0.14 (-0.80, 0.51)	0.663 (0.07)
** > 5 years current position**				
TRD (*N* = 18 obs.)	7.56 ± 1.76	7.33 ± 2.11	-0.22 (-1.04, 0.60)	0.585 (0.13)
RCDP (*N* = 15 obs.)	7.20 ± 1.52	6.87 ± 1.96	-0.33 (-1.23, 0.57)	0.456 (0.22)
**Physical demand**				
** ≤ 5 years current position**				
TRD (*N* = 28 obs.)	1.79 ± 1.66	1.46 ± 1.43	-0.32 (-0.93, 0.28)	0.293 (0.19)
RCDP (*N* = 35 obs.)	2.83 ± 2.42	2.91 ± 1.90	0.09 (-0.46, 0.63)	0.753 (0.04)
** > 5 years current position**				
TRD (*N* = 18 obs.)	6.06 ± 2.15	4.61 ± 2.15	-1.44 (-2.39, -0.50)	**0.004 (0.67)**
RCDP (*N* = 15 obs.)	4.93 ± 1.79	4.47 ± 1.68	-0.47 (-1.50, 0.57)	0.366 (0.26)
**Time demand**				
** ≤ 5 years current position**				
TRD (*N* = 28 obs.)	3.57 ± 2.50	3.50 ± 1.58	-0.07 (-1.20, 1.06)	0.901 (0.03)
RCDP (*N* = 35 obs.)	4.14 ± 2.80	3.23 ± 1.70	-0.91 (-1.92, 0.10)	0.076 (0.33)
** > 5 years current position**				
TRD (*N* = 18 obs.)	5.56 ± 2.41	4.67 ± 2.68	-0.89 (-2.09, 0.31)	0.143 (0.37)
RCDP (*N* = 15 obs.)	5.33 ± 2.38	4.13 ± 2.29	- 1.20 (-2.51, 0.11)	0.073 (0.50)
**Performance**				
** ≤ 5 years current position**				
TRD (*N* = 28 obs.)	3.64 ± 2.56	6.46 ± 2.35	2.82 (1.73, 3.91)	** < 0.001 (1.10)**
RCDP (*N* = 35 obs.)	3.83 ± 2.66	7.63 ± 1.72	3.80 (2.82, 4.78)	** < 0.001 (1.43)**
** > 5 years current position**				
TRD (*N* = 18 obs.)	6.67 ± 1.64	8.39 ± 0.85	1.72 (0.75, 2.70)	**0.001 (1.05)**
RCDP (*N* = 15 obs.)	5.53 ± 1.77	8.67 ± 1.50	3.13 (2.06, 4.20)	** < 0.001 (1.77)**
**Effort**				
** ≤ 5 years current position**				
TRD (*N* = 28 obs.)	4.75 ± 2.32	5.93 ± 2.28	1.18 (0.09, 2.27)	**0.035 (0.51)**
RCDP (*N* = 35 obs.)	4.09 ± 2.34	4.63 ± 1.80	0.54 (-0.43, 1.52)	0.270 (0.23)
** > 5 years current position**				
TRD (*N* = 18 obs.)	6.44 ± 2.55	5.78 ± 3.30	-0.67 (-1.61, 0.28)	0.160 (0.26)
RCDP (*N* = 15 obs.)	6.47 ± 1.30	6.00 ± 2.51	-0.47 (-1.50, 0.57)	0.365 (0.36)
**Frustration**				
** ≤ 5 years current position**				
TRD (*N* = 28 obs.)	3.96 ± 2.59	3.00 ± 1.78	-0.96 (-2.05, 0.12)	0.080 (0.37)
RCDP (*N* = 35 obs.)	4.11 ± 2.82	2.14 ± 1.54	-1.97 (-2.94, -1.00)	** < 0.001 (0.70)**
** > 5 years current position**				
TRD (*N* = 18 obs.)	4.83 ± 3.01	3.11 ± 2.95	-1.72 (-2.88, -0.56)	**0.005 (0.57)**
RCDP (*N* = 15 obs.)	4.53 ± 2.64	2.40 ± 2.23	-2.13 (-3.41, -0.86)	**0.002 (0.81)**

Effect size calculated by dividing the LS-mean difference by the SD at pre; ES interpreted as small (0.2), moderate (0.5), and large (0.8)

## Abbreviations

CHOA: Children’s Healthcare of Atlanta
CLT: Cognitive Load Theory
ED: Emergency Department
ES: Effect size
LS: Least square
IV: Intravenous
NASA TLX: National Aeronautics and Space Administration Task Load Index
RBAV: Read back and verification
RCDP: Rapid Cycle Deliberate Practice
RTs: Respiratory therapists
SbTT: Simulation-based team training
TRD: Traditional Reflective Debriefing
